# Retrospective analysis of salvage surgery for local progression of brain metastasis previously treated with stereotactic irradiation: diagnostic contribution, functional outcome, and prognostic factors

**DOI:** 10.1186/s12885-020-06800-w

**Published:** 2020-04-17

**Authors:** Koichi Mitsuya, Yoko Nakasu, Nakamasa Hayashi, Shoichi Deguchi, Takuma Oishi, Takashi Sugino, Kazuaki Yasui, Hirofumi Ogawa, Tsuyoshi Onoe, Hirofumi Asakura, Hideyuki Harada

**Affiliations:** 1 Division of Neurosurgery, Shizuoka Cancer Center, 1007, Shimo-nagakubo, Naga-izumi, Shizuoka, 411-8777 Japan; 2 Division of Diagnostic Pathology, Shizuoka Cancer Center, Shizuoka, Japan; 3 Radiation and Proton Therapy Center, Shizuoka Cancer Center, Shizuoka, Japan

**Keywords:** Brain metastasis, Prognostic factor, Radiation necrosis, Recurrence, Stereotactic irradiation, Surgery

## Abstract

**Background:**

Stereotactic irradiation (STI) is a primary treatment for patients with newly diagnosed brain metastases. Some of these patients experience local progression, which is difficult to differentiate from radiation necrosis, and difficult to treat. So far, just a few studies have clarified the prognosis and effectiveness of salvage surgery after STI. We evaluated the diagnostic value and improvement of functional outcomes after salvage surgery. Based on these results, we reconsidered surgical indication for patients with local progression after STI.

**Methods:**

We evaluated patients with brain metastases treated with salvage surgery for local progression from October 2002 to July 2019. These patients had undergone salvage surgery based on magnetic resonance imaging findings and/or clinical evidence of post-STI local progression and stable systemic disease. We employed two prospective strategies according to the eloquency of the lesions. Lesions in non-eloquent areas had been resected completely with a safety margin, utilizing a fence-post method; while lesions in eloquent areas had been treated with minimal resection and postoperative STI. Kaplan-Meier curves were used for the assessment of overall survival. Prognostic factors for survival were analyzed.

**Results:**

Fifty-four salvage surgeries had been performed on 48 patients. The median age of patients was 63.5 years (range 36–79). The median interval from STI to surgery was 12 months. The median overall survival was 20.2 months from salvage surgery and 37.5 months from initial STI. Primary cancers were lung 31, breast 9, and others 8. Local recurrence developed in 13 of 54 lesions (24%). Leptomeningeal dissemination occurred after surgery in 3 patients (5.6%). Primary breast cancer (breast vs. lung: HR: 0.17), (breast vs. others: HR: 0.08) and RPA class 1–2 (RPA 1 vs. 3, HR:0.13), (RPA 2 vs 3, HR:0.4) were identified as good prognostic factors for overall survival (OS) in multivariate analyses. The peripheral neutrophil-to-lymphocyte ratio (NLR) of ≤3.65 predicted significantly longer OS (median 25.5 months) than an NLR > 3.65 (median 8 months).

**Conclusion:**

We insist that salvage surgery leads to rapid improvement of neurological function and clarity of histological diagnosis. Salvage surgery is recommended for large lesions especially with surrounding edema either in eloquent or non-eloquent areas.

## Background

Stereotactic irradiation (STI) is a primary treatment option in the initial management of patients with brain metastases [1]. It is utilized alone, as adjuvant therapy after resection surgery, or in combination with whole brain irradiation (WBRT) [2–4]. One year after treatment STI has demonstrated local control rates of 70 to 90% when used alone for metastases smaller than 3 cm, irrespective of primary cancer histology [2, 5–7]. Late tumor recurrence and radiation injury are relatively rare events following STI. With improved local and systemic management in recent years, patients with brain metastases now survive for longer periods. These post-STI patients are showing late clinical deterioration with locally progressive mass lesions.

This local progression can possibly be any of these three pathological conditions: local recurrence of metastasis, radiation necrosis, or a combination of both. The clinical and imaging features of local recurrence and radiation necrosis show considerable overlap. Even modern multimodal examinations demonstrate insufficient specificity in this context.

Patients with local progression are managed by either WBRT, repeat STI or surgery [1, 8]. Each treatment modality has limitations: WBRT may cause exhaustion and frequent decline in higher cortical function, and is avoided or deferred unless it is necessary to control multiple brain lesions; repeat STI to the local progression leads to increased toxicity to the brain by a high-dose re-irradiation, especially if there is radiation necrosis or combination of recurrence and necrosis [9]; surgical resection is not a much-published treatment modality for post-STI local progression.

Surgery is recommended for accessible lesions, especially if symptoms are progressive. Surgery immediately reduces the mass effects of these lesions and enables pathological confirmation. However, most reports on salvage surgery consist of small cohorts and heterogeneous groups. Lack of published reports on salvage surgery could be because these are cancer patients with limited life expectancy, who may assume that control would be insufficient, and treatment would lead to excessive morbidity.

Although many patients suffering from local progression are seen in the recent years, few reports provide data on the indications for surgery and surgical techniques.

Unlike brain metastases before treatment, local progression lesions after STI show unclear margins, leading to a technical difficulty in surgical resection. Additionally, these lesions have a complex intermingling of cancer cells with injured brain or necrotic tissue [10–12]. Therefore, neurosurgeons employ two different approaches to avoid recurrence or cancer cell dissemination: (1) resection with safety margins around the local progression, or (2) intended minimal resection along the borders with planned postoperative STI. Since 2003, we have employed a prospective strategy with a safety margin, utilizing a fence-post method for local progressions in non-eloquent areas, and a minimal resection accompanied by postoperative STI for progressions in eloquent areas.

The aim of this study was to retrospectively evaluate our surgical resection techniques to treat locally progressive lesions, for feasibility, efficacy in functional outcome by Karnofsky performance status (KPS) changes, and indications. Particularly, we examined whether the peripheral blood neutrophil-to-lymphocyte ratio (NLR) before surgery was a prognostic marker for salvage surgery, as NLR is a useful prognostic marker in upfront surgery for brain metastases [13].

## Methods

Analysis of the medical records was performed after approval by the Institutional Research Ethics Board of the Shizuoka Cancer Center (30-J53–30–1-3). Our ethics board waived the requirement for written informed consent for this retrospective observational study. After anonymizing the data, we reviewed the neurosurgical reports, survival data, corresponding plans of STI, images, and independent pathological reports by the two treating neurosurgeons (K.M. and Y.N.).

### Patients

From June 2004 to July 2019, brain metastases in 827 patients were treated with stereotactic radiosurgery (SRS) or stereotactic radiotherapy (SRT) at our institute. We identified 48 patients with 54 lesions that required salvage surgery for local progression of brain metastases after stereotactic irradiation (STI: SRS or SRT) between October 2002 and July 2019. We included patients who underwent salvage surgery for local progression at our institute, even though STI had been performed at another institute. Five patients had undergone SRS twice for the same brain metastases. Thirty-nine patients had received treatments before STI: chemotherapy alone (*n* = 31), and WBRT+ chemotherapy (*n* = 8).

We evaluated the radiological and clinical data at follow-up clinic visits. Patients’ general condition was assessed on the day of surgery and 1 month after surgery, using the Karnofsky Performance Score (KPS). MR imaging was performed every 3 months after STI unless the treating oncologist/ neurosurgeon had requested more frequent imaging.

Routine radiological data of patients with brain metastases included unenhanced T2, FLAIR, T1 and diffusion-weighted images, ADC-maps, and contrast-enhanced T1-images. Local progression was suspected at the site of STI if MR imaging showed regrowth of an enhanced mass months after any decrease in size. In selected cases with suspected local progression, we added perfusion-weighted CT scans with CBV-maps using a 320-row CT scanner (Aquilion ONE, Canon Medical Systems) [14]. We did not conduct positron emission tomography scans.

### STI as initial treatment for brain metastases

STI alone was used to treat 46 lesions, and STI + WBRT was used to treat 8 lesions. Radiation oncologists performed STI using the linear accelerator stereotactic radiosurgery system (m3, Brain LAB, or TrueBeam STx, Varian). Dose selection was made on the basis of tumor volume, multiplicity, location or eloquency, previous fractionated radiotherapy, and the prescription isodose guidelines of RTOG 90–05.

### Indications for salvage surgery

Local progression, diagnosed on the basis of perfusion-weighted CT scans, contrast-enhanced MR images, and clinical status, guided the treatment decisions made by our multidisciplinary cancer board. Patients were considered eligible for salvage surgery if (1) they were in good systemic condition with controlled or stable disease (2) the lesion was surgically accessible, (3) rapid progression mandated an early decompression, and (4) symptomatic mass effect unresponsive to maximal medical therapy including corticosteroids, (5) no other treatment options remained except surgery. Patients with an unknown primary cancer were also considered eligible for resection surgery.

### Surgical techniques

**Fig. 1:**
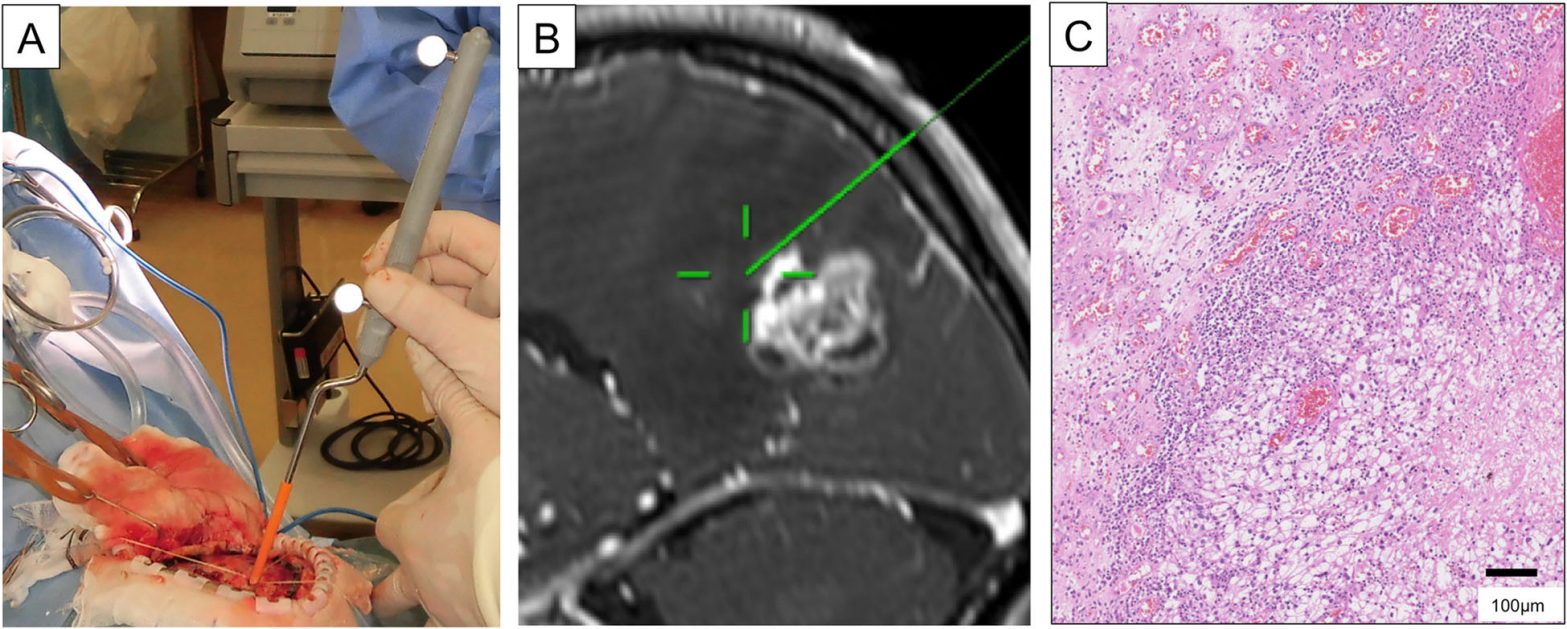
Local progression of brain metastasis from renal cell carcinoma previously treated with SRS was depicted at navigation surgery **a**, with an enhanced mass with navigation trajectory in the occipito-parietal region **b**. A histopathological picture of the border zone **c**. The margin between the tumor and the brain was collapsed. Hematoxylin and eosin staining showed neutrophil infiltration at the tumor margin, gliotic changes and a large number of microvessels in the surrounding brain tissue (× 10)

During surgery, macroscopic borders between the brain and the local progression are unclear because the irradiated tumor, necrotic tissue, and the gliotic brain have similar gross appearances [11]. To preserve the maximum brain function, surgical dissection borders were planned in two ways. In the eloquent areas of the brain, we dissected the mass lesion in its border, allowing for minimum resection. and gave postoperative SRT or WBRT if necessary. In the non-eloquent areas, we used the navigation-guided fence post method to remove lesions totally, along with abnormal surrounding tissue and surrounding safety margins, without any adjuvant irradiation [15]. Figure 1 shows a representative case of local progression during navigation surgery (Fig. 1 A). An enhanced mass is seen in the occipito-parietal region with navigation trajectory (Fig. 1 b), and the histopathological picture of the border zone is shown (Fig. 1 c).

### Statistical analysis

Overall survival (OS) was calculated from the date of salvage surgery for local progression to the date of death due to any cause. Statistical analysis was performed using JMP (version 11, SAS Institute), and data for OS were analyzed using the Kaplan-Meier method. OS values were compared using the log-rank test. Frequency analysis was performed using Fisher’s exact test. *P*-value ≤0.05 was considered statistically significant.

## Results

### Patient and tumor characteristics

A total of 54 salvage surgeries were performed for local progression in 48 patients, including twice for the same lesion in two patients, and for two different lesions in three patients (Table 1). If this 18-years observation period is divided into halves, salvage surgeries increased in number from 20 cases in the first 9 years (mean: 2.2 cases /year), and to 34 cases in the recent 9 years (mean: 3.8 cases/year). The series included 32 men and 16 women as a total number of patients with a median age of 63.5 years (range 36–79 years) at the time of salvage surgery. Primary cancers were lung in 31 (64.6%), breast in 9 (18.8%) and others in 8 (16.6%) patients (renal cell carcinoma 2, melanoma 2, colon cancer 2, uterus 1, and mediastinum carcinoma 1). The median diameter of the enhanced lesions was 35 mm (range 19–58 mm).

**Table 1:** Patient characteristics

	No. (%)
Patients	48
Lesions	54
Median overall survival from surgery (months)	20.2
Median age at surgery (years)	63.5
Median time from STI to surgery (months)	12
Gender	
Male	32 (66.7)
Female	16 (33.3)
Primary cancer	
Lung	31 (64.6)
Breast	9 (18.8)
Others	8 (16.6)
Location	
Supratentrial	47 (87)
Infratentrial	7 (13)
Neurological deficit	
Yes	36 (67)
No	18 (33)
RPA classification at surgery	
Class1	7 (13)
Class2	18 (33)
Class3	29 (53)
Radiotherapy before salvage surgery	
STI	46 (85)
SRS	24 (44.5)
SRT	17 (31.5)
Repeated STI	5 (9)
WBRT+STI	8 (15)
Surgical method	
Minimum resection	26 (48)
with radiotherapy	12 (22)
without radiotherapy	14 (26)
Resection with free margine	28 (52)
Pathological diagnosis	
Tumor recurrence + Necrosis	47 (87)
Radiation necrosis alone	7 (13)
Extent of resection	
Gross total removal	48 (89)
Subtotal removal	6 (11)
KPS before surgery	
90–100	18 (33)
70–80	5 (9)
50–60	20 (37)
30–40	11 (20)
0–20	0 (0)
KPS after surgery	
90–100	24 (44)
70–80	16 (30)
50–60	10 (18.5)
30–40	3 (5.5)
0–20	1 (2)
Radiotherapy after salvage surgery	
Yes	20 (38)
WBRT	10 (18.5)
STI	10 (18.5)
No	34 (62)
Treatment period	
2002–2010	19 (40)
2011–2019	29 (60)

Twenty patients had received systemic therapy after STI, and before local progression was detected (*n* = 8 for cytotoxic chemotherapy, *n* = 9 for targeted therapy, *n* = 3 for immuno-checkpoint inhibitor therapy). In 29 of the 54 lesions (53.7%), there was no systemic metastasis at the time of salvage brain surgery, local brain progression being the only metastatic complication. After salvage surgery, 18 patients required systemic therapy (*n* = 6 for cytotoxic chemotherapy, *n* = 9 for targeted therapy, and *n* = 3 for immuno-checkpoint inhibitor therapy).

Twenty-six lesions (48%) were removed with minimum resection technique as they were in the eloquent areas, and 28 lesions (52%) were removed with surrounding free margin for safety (Table 1). Twelve of the 26 lesions treated with minimal resection (46.1%) were treated with either postoperative adjuvant radiosurgery or WBRT. The median interval from initial STI to the salvage surgery was 12 months (range 0.5–125.3 months).

### Survival outcome

**Fig. 2:**
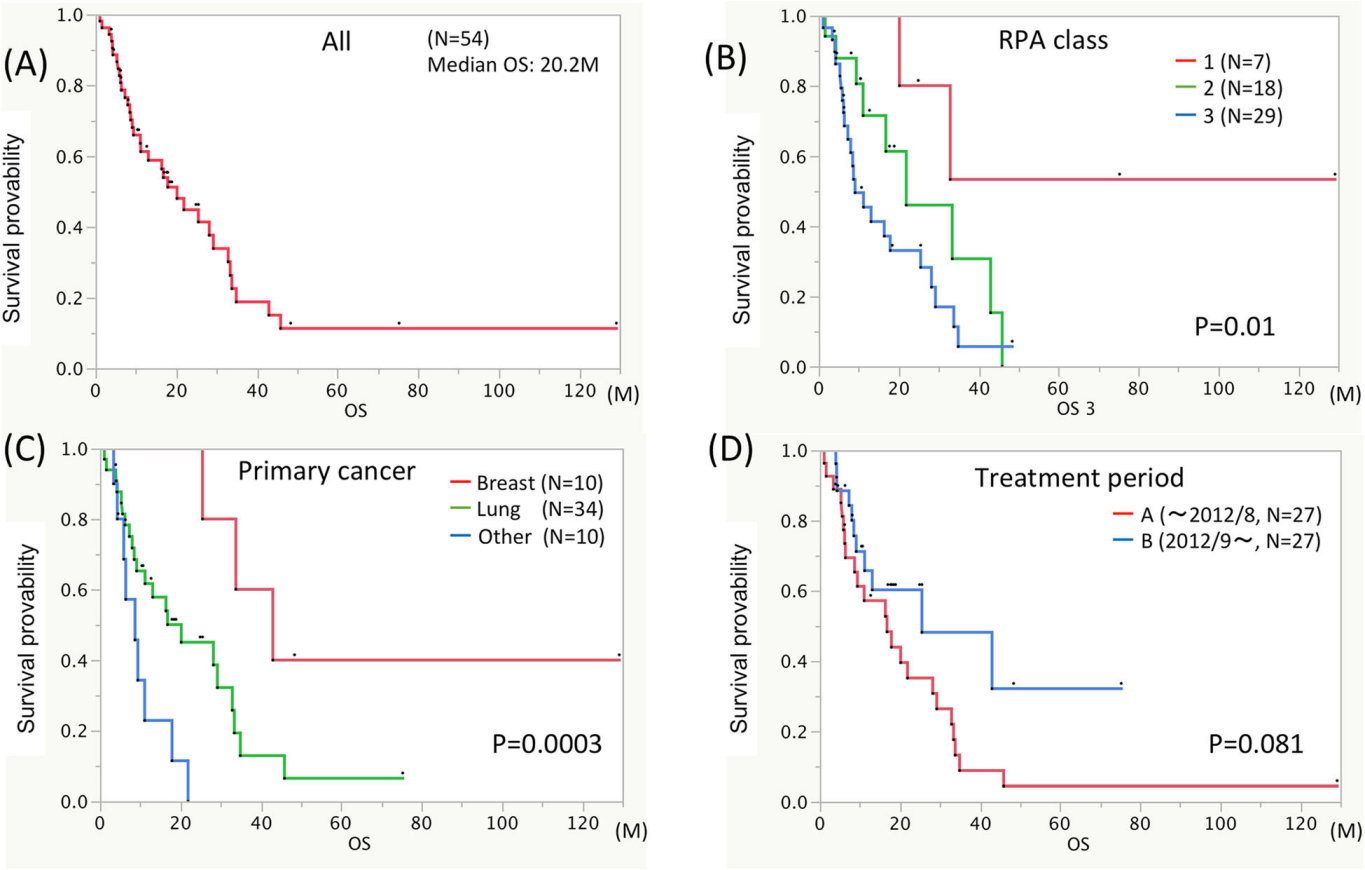
Median OS was 20.2 months from salvage surgery **a**. RPA classes **b** and primary cancer histology **c** showed a significant correlation with OS on univariate analysis. Patients showed a trend towards longer survival in recent years **d**

Median OS after salvage surgery was 20.2 months (Fig. 2 a). In the alive patients, the probability of local failure after salvage surgery did not reach a median after following up for 11.2 (median) months (range 0.9–129.2 months). Median OS after upfront STI was 37.5 months in all the patients (*n* = 48), 37.5 months in patients with either pure or combination histological recurrence (*n* = 42) and 40.3 months in patients with pure histological necrosis (*n* = 6) (*P* = 0.396).

We conducted univariate and multivariate analyses for survival despite the small n value for patients and events (Table 2). Tumor location, primary cancer histology and RPA classes correlated significantly with survival (Fig. 2). Multivariate analyses demonstrated that breast cancer, and RPA classes 1 or 2 versus class 3 correlated significantly with longer survival (Table 2). We did not exclude patients of RPA class 3 and low KPS from being candidates for resection surgery, as they comprised a large percentage (53% RPA class 3 and 57% low KPS) of our cohort (Table 1). Local recurrence and leptomeningeal dissemination did not correlate with the selection of surgical technique. The local recurrence rate was 24% (13/54 cases) 1 year after resection surgery, and leptomeningeal dissemination was 5.6% at the end of the observation period (Table 3).

**Table 2:** Univariate and multivariate analysis of patient and treatment factors for survival

Tested Variable	Number (%)	Univariate		Multivariate			
		median OS (M)	*P* value	Tested Variable	HR	95%CI	*P* value
Age at surgery (median; 63.5)							
< 63-years	21 (43.8)	29.2	0.4				
≥ 63-years	27 (56.2)	16.8					
Median time from STI to surgery (12mo)							
≥ 12 months	22 (45.8)	32.9	0.13				
< 12 months	26 (54.2)	13.1					
Pathological diagnosis							
Radiation necrosis alone	6 (12.5)	22.8	0.76				
Tumor recurrence + Necrosis	42 (87.5)	20.2					
Gender							
Female	16 (33.3)	29	0.11				
Male	32 (66.7)	16.8					
**Location**				**Location**			
Supratentrial	42 (87.5)	25.5	**0.016**	Supra- vs Infratentrial	0.5	0.18–1.52	0.2
Infratentrial	6 (12.5)	9.1					
**Primary cancer**				**Primary cancer**			
Breast	9 (18.8)	43	**0.003**	**Breast vs Lung**	**0.2**	**0.036–0.55**	**0.002**
Lung	31 (64.6)	16.8		**Breast vs Others**	**0.1**	**0.013–0.34**	**7E-04**
Others	8 (16.6)	9.4		Lung vs Others	0.5	0.2–1.2	0.12
Neurological deficit							
No	15 (31.3)	21.9	0.56				
Yes	33 (68.7)	16.8					
**RPA**				**RPA**			
Class 1	6 (12.5)	NR	**0.023**	**Class 1 vs 3**	**0.1**	**0.02–0.51**	**0.002**
Class 2	18 (37.5)	21.9		**Class 2 vs 3**	**0.4**	**0.16–0.95**	**0.042**
Class 3	24 (50)	11.2		Class 1 vs 2	0.3	0.05–0-1.35	0.13
Surgical technique							
Minimum resection	23 (48)	25.5	0.7				
Resection with free margine	25 (52)	17.9					
Extent of resection							
Subtotal removal	6 (12.5)	28.2	0.98				
Gross total removal	42 (87.5)	20.2					
Radiotherapy after salvage surgery							
Yes	15 (31.3)	20.2	0.38				
No	33 (68.7)	16.8					

*NR not reached

**Table 3:** Surgical technique and outcome

	All lesions	Minimum resection +/− SRT	Resection with free margin	*P* value
Number of lesions	54	26	28	
Local recurrence	13 /54 (24%)	9 /26 (34.6%)	4 /28 (14.3%)	0.07
Leptomeningeal Dissemination	3 /54 (5.6%)	3 /26 (11.5%)	0 /28 (0%)	0.105

*Fisher’s exact test

**Fig. 3:**
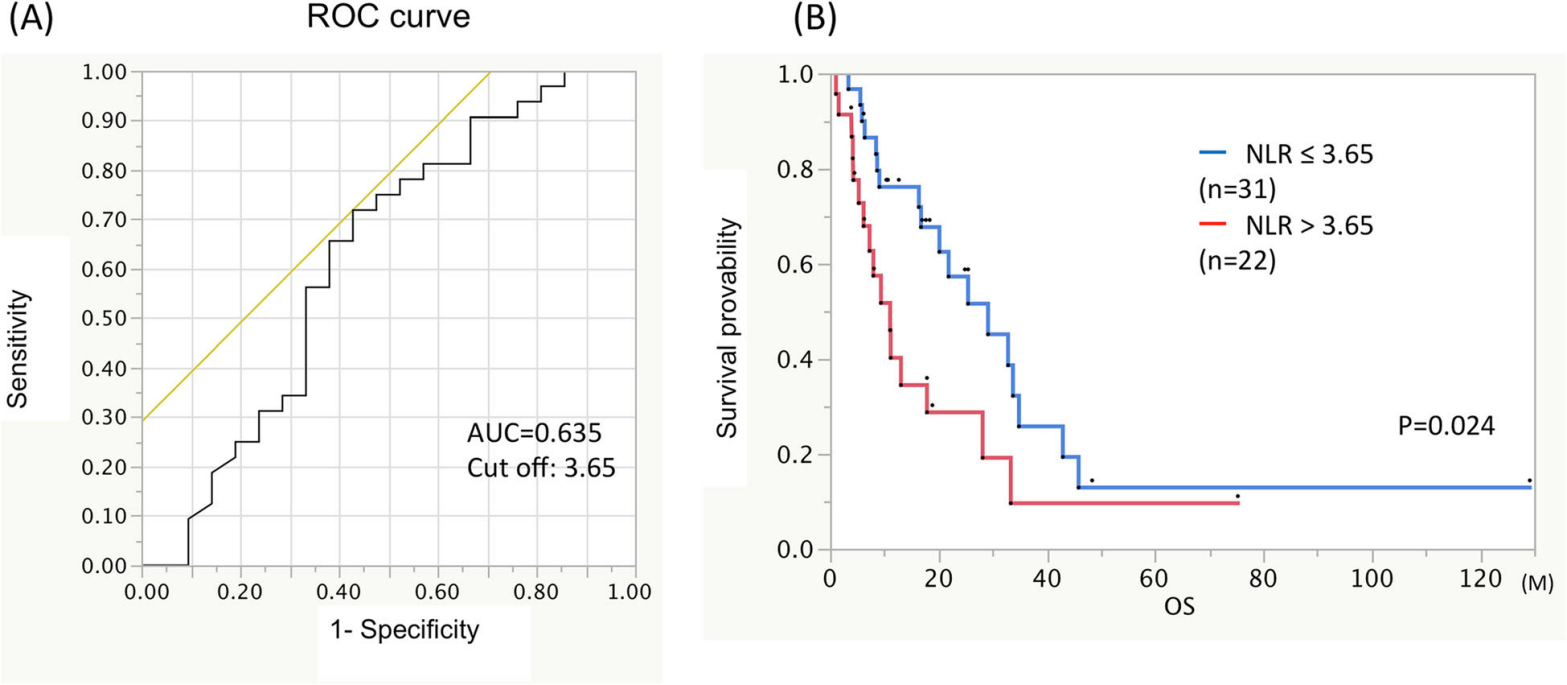
NLR was evaluated by Receiver Operating Characteristic curves, showing a maximum area under the curve of 0.635 **a**. Kaplan-Meier survival curves of patients with low preoperative NLR (≤ 3.65) were compared with those with a high NLR (> 3.65). The median OS was significantly longer in patients with a low NLR (25.5 months) than those with a high NLR (8 months) **b**

Preoperative NLR was evaluable in 53 of 54 cases, except for one with a technical problem. The optimum NLR threshold value was identified as 3.65 for survival time on an ROC curve (Fig. 3a). The patients with an NLR ≤ 3.65 showed significantly longer median OS than those with an NLR > 3.65, 25.5 months (*N* = 31) versus 8 months (*N* = 22) respectively (Fig. 3b, *p* = 0.024).

### KPS changes

**Fig. 4:**
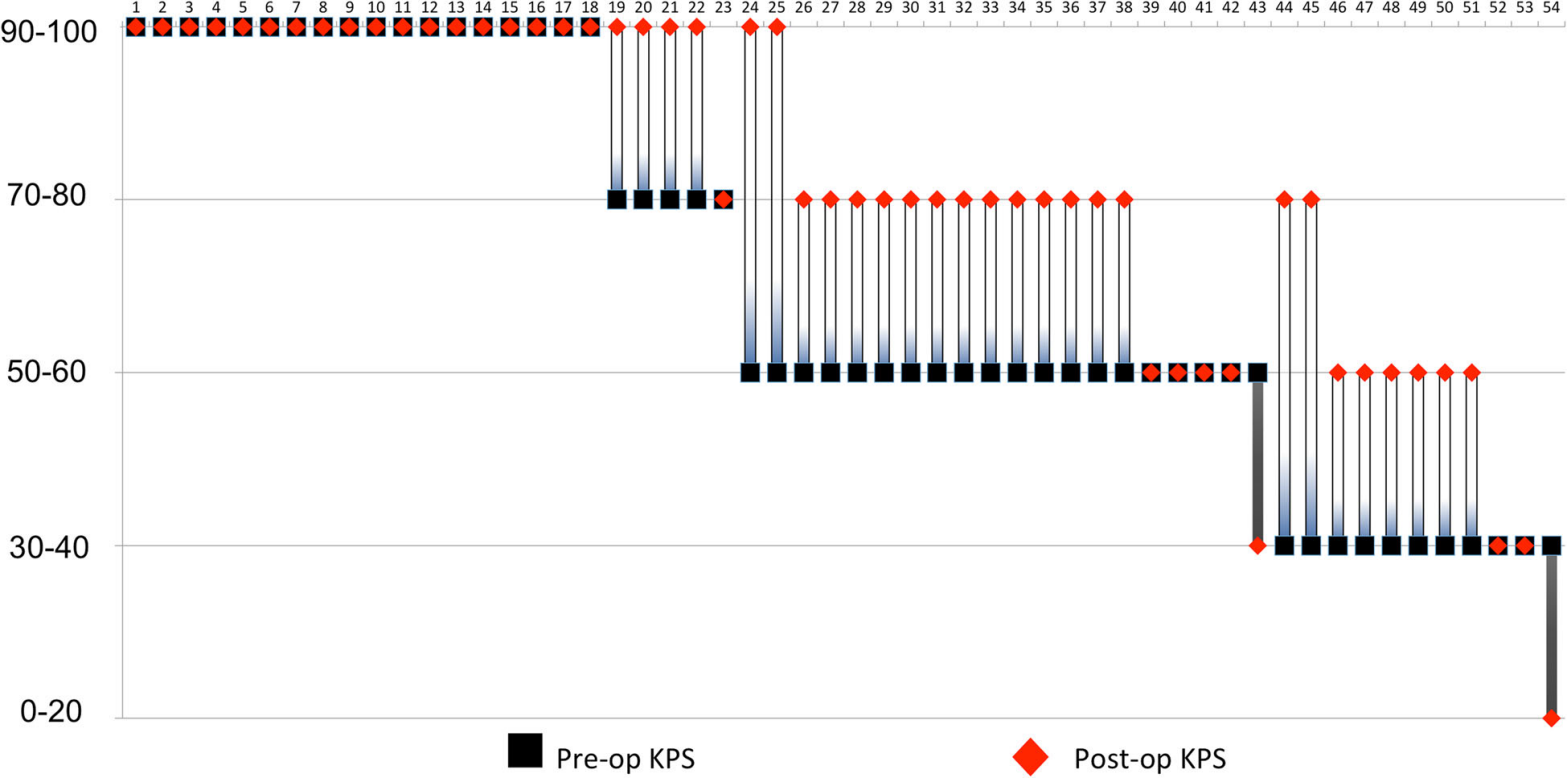
Changes of KPS in individual cases. Among 36 cases having lower KPS due to neurological deficits before surgery, 27 (75%) cases showed improved KPS, seven (19.4%) showed no change, and two (5.6%) deteriorated

Figure 4 demonstrates changes in KPS of individual patients. Preoperatively, 31 of 54 cases (57.4%) presented KPS 30–60%, and 23 of 54 cases (42.6%) presented KPS 70–100%. Twenty-three of 31 cases (74.2%) with worse KPS (30–60%) showed improvement of KPS postoperatively. Among 36 of 54 (66.7%) cases with neurological deficits before surgery, 27 (75%) cases showed improvements, seven (19.4%) showed no change, and two (5.6%) deteriorated.

### Adverse effects of salvage surgery

No mortality occurred within 30 days of salvage surgery in our cohort. Two patients (3.7%) showed deterioration in their KPS, due to the progression of primary cancer after brain surgery, not due to neurological morbidity (Fig. 4).

## Discussion

STI is the primary treatment for newly diagnosed brain metastases, especially if they are small and inaccessible. Local progression of brain metastases after upfront treatment with STI is seen more often now because of better survival of these patients, and the demand for salvage treatment is now more than ever [16]. In our study, salvage surgery was done for 20 cases in the first 9 years, and for 34 cases in the recent 9 years.

Median OS after upfront STI was 37.5 months for all 48 patients, 37.5 months for recurrences with pure or combined histology (*n* = 42), and 40.3 months for patients with pure radiation necrosis (*n* = 6) (*p* = 0.13). Median OS noted in previously published studies was much lower at 7.5 to 19 months (Table 4) [10, 11, 17–20]. The longer median survival in our study can be attributed to the development of systemic therapy, improved care of cancer patients, and advances in surgical techniques.

**Table 4:** Reports of surgical resection for recurrent/ progressive brain metastases after STI

First Author, Year [ref]	Number of patients	Surgical lesion	RPA class population I/2/3 (%)	Previous treatment	Median tumor volume or maximum diameter	Rates of radiation necrosis alone (%)	Median survival (months)	# Pts with local recurrence after surgery (%)	# Pts with dissemination after surgery (%)	Surgical mortality (%)
Vecil, 2005 [17]	61	74	13/72/15	SRS	NR	6/74 (8)	11.1	13/ 74 (17.6)	NR	3.0
Truong, 2006 [10]	32	32	13/87/0	SRS	1.5 cc	4/32 (12.5)	8.9	9/ 32 (28)	NR	3.0
Kano, 2009 [18]	58	58	38/52/10	SRS	15.5 cc	0 (0)	7.6	18/ 58 (31)	NR	1.7
Jagannathan, 2010 [11]	15	15	NR	SRS	7.5 cc	5/15 (33.3)	11.3	0/ 15 (0)	NR	NR
Schackert, 2013 [19]	67	67	24/46/30	SRS	NR	NR	7.5	21/ 67 (31.3)	NR	NR
Telera, 2013 [20]	15	16	50/25/25	SRS	3 cm (1.5–4.5)	7/16 (50)	19.0	0/16 (0)	NR	0.0
Present study, 2019	48	54	13/31/56	SRS or SRT	3.5 cm	7/54 (13)	20.2	14/54 (25.6)	3/54 (5.6)	0.0

*NR Not reported,

# *Pts* patients

Overall survival of patients with brain metastases also depends on the outcome of systemic primary cancers. However, these patients’ functional outcome depends essentially on the CNS lesions. The goal of CNS treatment is to delay neurological progression long enough to facilitate the treatment of primary systemic metastases. Therefore, we should consider functional outcomes along with OS while considering treatment options for individual patients with brain local progression [21]..

Treatment decisions for brain local progression should be based on the functional status, lesion volume and surrounding edema, previous treatments, and the type of primary cancer [21]. Surgical removal, repeated in-field radiosurgery, WBRT, systemic therapy, or a combination of these can be chosen, depending on these factors.

Surgery remains an effective salvage treatment option with two important benefits: immediate reduction of the mass effects and histological confirmation. Salvage surgery led to overall local control rates of 69 to 100%, and a median OS of 7.5 to 20.2 months, even for large lesions (Table 4).

Genetic and molecular information of cancer cells has become a recent clinical focus, because of reports showing drafting and variation of genetic markers in metastatic cancer cells [22, 23]. Pathological analysis of surgical specimens may present new opportunities for targeted therapy.

In our study, histological examination revealed that 13% of the local progression lesions were radiation necrosis alone, and 87% were a heterogeneous combination of necrosis and cancer cells. Previous studies have shown pure radiation necrosis in 0–50% of lesions obtained by salvage surgery (Table 4). The preoperative differential is not always easy, because both radiation necrosis and tumor recurrence present similar clinical and radiological findings, and a majority of these lesions comprise a combination of necrosis and cancer cells [20, 24]. Without histological confirmation, repeated irradiation can lead to deterioration of necrosis and brain edema.

Retrospective studies on the one-year outcome of repeated radiosurgery for local progression reported that local control was 61 to 88%, and OS was 37 to 90.6% [25–27]. Toxicity was reported as overall necrosis rates 9.2 to 19% after the repeated SRS or SRT [25, 26]. Necrosis after repeated STI was shown to be significantly associated with the irradiated volume and the cumulative dose [16]. For large lesions with surrounding edema, repeat SRS may lead to a risk of local progression as the majority of these lesions consist of a combination of radiation necrosis and cancer cells, surrounded by the injured brain. In our study, repeated in-field STI was used for cancer cells remaining after minimal resection, in eloquent areas only. The risk of radiation necrosis due to repeated STI is reduced after decompressive surgery, because of less target volume and less involved normal tissue volume in the intended field.

We treated patients even if their prognostic scores were poor. 50% of patients in our cohort were RPA class 3, whereas these advanced class patients were maximum of 30% in previous studies (Table 4). RPA classes were significantly correlated with the median OS (Table 2). Interestingly, the median OS of the whole cohort in our study was longer than the median OS in previous studies. We offered surgical resection to patients with lower KPS when we expected that neurological improvement would lead to a better general condition. Very little has been reported about postoperative functions after salvage surgery. In our study, 23 of 54 cases showed improvement in KPS after salvage surgery (Fig. 4). Sixteen patients returned to their systemic therapy for primary cancer soon after recovering from salvage surgery.

Although the numbers of patients presenting with local progression are increasing in recent years, most studies on salvage surgery, published before 2014, comprised of small cohorts (Table 4). This lack of recent data may be due to patients assuming upon a high risk of surgical resection and general anesthesia in heavily treated patients with brain metastases.

Surgical resection is safe for local progression after STI in selected patients. Surgical mortality rates range from zero in Telera’s series [20] and ours, to 3% in the series of Vecil [17] and Truong [10] (Table 4). Although these surgical mortality data are of patients with heavily treated cancer, the rates were not worse than the data of general patients who underwent craniotomy for brain tumors. If we compare surgical morbidity data, previous studies reported little regarding postoperative neurological function, KPS or QOL. In our study, for lesions in eloquent areas, the minimum resection technique was used to preserve surrounding normal tissue, to achieve the best possible neurological function and KPS. Postoperative STI was used only if active cancer cells had invaded the surrounding brain and leptomeningeal layers. This combination strategy was as effective as resection with free margin and no postoperative radiation, in achieving local control and preventing dissemination (Table 3). This result emphasizes that proper selection of surgical technique contributes to a better functional outcome as well as curability. Although resection with free margin is an effective surgical technique for metastatic lesions with tissue invaded by cancer cells, it can be employed only in non-eloquent areas.

Two of our patients showed a deterioration of KPS, which occurred due to unexpected and rapid progression of primary cancer after salvage surgery, and clearly not due to neurological progression. Preoperative thorough assessments are complex challenges, especially in patients with heavily treated cancer. In this study, NLR has proven itself once again as a reliable marker to evaluate the general condition of patients with local progression. Previously, this was indicated in our study on upfront surgery for brain metastases [13]. NLR is a simple peripheral blood sample method and would help in the decision-making for resection surgery. We have various assessment tools to predict the survival prognosis of patients with brain metastases [28, 29]. However, we do not yet have any assessment tools to predict the functional outcome of brain metastases patients after local or systemic treatments. Long-term observation of large cohorts is necessary to predict the functional outcome of brain metastases patients.

Our study has some limitations, as it was a retrospective study of therapeutic outcomes with surgical resection only. We had a concrete plan to select patients for resection, and observe in the postoperative period with MR imaging. Planning for a control group was not ethical for this relatively rare, progressive and devastating condition. The patient cohort was small because this is a study in a single cancer center. A majority of the patients had primary cancers of lung and breast, but only a few had melanoma as a primary. This is an ethnical tendency that might taint a direct comparison of our outcomes with previous studies on Western populations.

## Conclusion

Our retrospective study shows that salvage surgery after upfront STI is safe and gives good functional outcome in patients with local progression of brain metastases. Histologically, a majority of these lesions were a combination of necrosis and cancer cells. Additionally, peripheral blood NLR proved to be a reliable marker to assess the general condition of patients who were heavily treated, and presented with local progression.

We insist that surgical resection leads to rapid improvement of neurological function, and aids histological diagnosis. Salvage surgery is recommended for large lesions, especially if there is surrounding edema, in both eloquent and non-eloquent areas. Minimal resection technique combined with postoperative STI is recommended for lesions in the eloquent areas and removal with safety margin around the local progression for lesions in non-eloquent areas.

## Data Availability

The datasets analyzed during the current study are available from the corresponding author on reasonable request.

## Abbreviations

STI: Stereotactic irradiation
WBRT: Whole brain radiation therapy
NLR: Neutrophil-to-lymphocyte ratio
SRS: Stereotactic radiosurgery
SRT: Stereotactic radiotherapy
KPS: Karnofsky performance score
FLAIR: Fluid -attenuated inversion recovery
ADC: Apparent diffusion coefficient
CBV: Cerebral blood volume
RTOG: Radiation therapy oncology group
RPA class: Recursive partitioning analysis
CNS: Central nervous system
OS: Overall survival
